# Novel DNA variation of *GPR54* gene in familial central precocious puberty

**DOI:** 10.1186/s13052-019-0601-6

**Published:** 2019-01-11

**Authors:** Nosrat Ghaemi, Martha Ghahraman, Samaneh Noroozi Asl, Rahim Vakili, Fatemeh Fardi Golyan, Meysam Moghbeli, Mohammad Reza Abbaszadegan

**Affiliations:** 1 Department of Pediatric Endocrinology and Metabolism, Imam Reza Hospital, School of Medicine, Mashhad University of Medical Sciences, Mashhad, Iran; 2 Immunology Research Center, Mashhad University of Medical Sciences, Mashhad, Iran; 3 Medical Genetics Research Center, Mashhad University of Medical Sciences, Mashhad, Iran; 4 Department of Pediatric, Valiasr Hospital, Birjand University of Medical Sciences, Birjand, Iran; 5 Department of Modern Sciences and Technologies, Faculty of Medicine, Mashhad University of Medical Sciences, Mashhad, Iran

**Keywords:** Familial precocious puberty, *GPR54* gene, Kisspeptin, Novel SNP

## Abstract

**Background:**

Puberty can be considered the end point of a maturation process which is defined by the dynamic interactions of genes and environmental factors during prenatal and postnatal development. Kisspeptin/G protein-coupled receptor-54, is as an essential gatekeeper and regulator of GnRH neurons, and a key factor in initiation of puberty. Loss and gain of functional mutations in the GPR54 gene are associated with hypogonadotropic hypogonadism and precocious puberty, respectively. This study was designed to evaluate variations of GPR54 in familial precocious puberty.

**Methods:**

Genomic DNA was extracted from peripheral whole blood of 25 subjects with familial precocious puberty. Coding exons 1–5 of the *GPR54* gene were amplified by polymerase chain reaction (PCR) and the PCR products were purified and sequenced. DNA sequences were compared to the human GenBank GPR54 sequence using Sequencher sequence alignment software.

**Results:**

We detected three different Single Nucleotide Polymorphisms (SNPs) in *GPR54*: rs10407968 (24A > T) in 13 subjects (52%); rs3050132 (1091 T > A) in 16 subjects (64%), and a novel polymorphism (492C > G) in one subject (4%), while three subjects (12%) had no SNPs. No mutations were found in the GPR54 gene.

**Conclusions:**

Regarding the presence of SNPs in 88% of the subjects in this study, it is likely a relationship exists between the SNPs of the *GPR54* gene and familial precocious puberty. Further research is needed to investigate this possibility, and potential functional effects of these polymorphisms.

## Background

Puberty is a complex biological process that can be influenced by genetic, nutritional, environmental, and socioeconomic factors. Puberty is initiated by activation of pulsatile hypothalamic gonadotropin-releasing hormone (GnRH) followed by pituitary gonadotropin secretion and gonadal steroid production. Precocious puberty is defined as the development of secondary sexual characteristics before the ages 8 years in girls and 9 years in boys which is classified as gonadotropin-dependent or independent. Gonadotropin-dependent (central) precocious puberty has a striking predominance among girls, and most of the cases are considered idiopathic. Genetic factors have important roles in the time of pubertal onset. In recent studies autosomal dominant transmission has been reported with incomplete sex-dependent penetrance [1], and several gene mutations that influence puberty have been identified [2]. Activating mutations in the G protein-coupled receptor, and its ligand, kisspeptin, products of the *GPR54* (NM-032551) and KiSS1 genes, respectively, were identified recently as causes of central precocious puberty [3–5]. GnRH secretion mediated by kisspeptin activation of *GPR54*, also referred to as KiSS1R, AXOR12, and OT7T175, which is responsible for the onset of puberty [6, 7]. The *GPR54* gene, located in the vicinity of 19q13.3 and approximately 3 kb in length, has five exons interrupted by four introns and contains an open reading frame (ORF) of 1197 bps encoding a protein of 398 amino acids. *GPR54* is evolutionarily conserved in mammals with the similarities of *GPR54* among goat, pig, bovine, and sheep higher than those of other mammalian species, such as human, rat, and mouse [8, 9]. Kisspeptin is a relatively recent discovery in hormonal control of reproduction [10, 11]. Activation of *GPR54* is apparently sufficient to trigger the neuro-endocrine events leading to the onset of puberty [12]. The aim of present study was to assess the incidence of genetic variations in *GPR54* gene for the first time in Iranian familial cases with precocious puberty. We evaluated 225 patients with central precocious puberty and selected 25 subjects with familial central precocious puberty for *GPR54* gene mutation studies.

## Methods

We assessed 25 subjects with familial central precocious puberty (CPP) who were recruited from the pediatric department of Imam Reza hospital. The study protocols were approved by the Ethics Committee for Human Research of the Mashhad University of Medical Sciences. CPP was diagnosed according to the following criteria: Breast development before the age of 8 years in girls and testicular enlargement before the age of 9 years in boys were assessed by the pediatric endocrinologist according to the Tanner Staging System. Advanced bone age more than one year above the chronological age was measured via the Greulich and Pyle method. Increased gonadotropin levels including luteinizing hormone (LH) greater than 4.5 mIU/mL, follicle stimulating hormone (FSH) greater than7 mIU/mL, and LH/FSH ratio greater than one were observed. The Immune Radiometric Assay method (IRA) was used to measure hormone levels. GnRH stimulation tests were performed according to clinical findings and hormone levels; they were compared to age-related reference levels: estradiol greater than 20 pg/mL and testosterone greater than 300 ng/dL. Gonadal sonographic findings compatible with pubertal stage: in females, uterus and ovary sizes greater than 40 mm and 25 mm, respectively, and in males, testis lengths and volumes greater than 30 mm and 3 cc, respectively. Inclusion criteria included presence of another CPP case in the family, age of the first menstruation under 10 years old in the first-degree relative’s female, and the age of the first complete face shaving before 13 years old in the first-degree relative’s male. To exclude possible brain abnormalities, magnetic resonance imaging was performed in females under five years and all males. Moreover, the cases with adrenal hyperplasia, CNS tumor, and ovarian cyst were also excluded from the present study. After the confirmation of CPP, we selected 25 subjects with familial CPP (Table 1). Genomic DNA was extracted from peripheral whole blood of these subjects [13]. The coding exons 1–5 and intron/exon boundaries of *GPR54* were amplified by polymerase chain reaction (PCR) using a combination of five primer pairs. PCR products were assessed for the size and purity by separation on 2% agarose gel electrophoresis and products were purified using a DNA Gel Extraction Kit (INVITEK) according to the manufacturer’s protocol. Bidirectional sequencing of purified amplicons was performed using an Applied Biosystems ABI 3730 XL automated DNA Sequencer. The sequences were compared to the human GenBank sequence for *GPR54* using Sequencher sequence alignment software (Version 4.10.1).

**Table 1:** Clinical and Homoral features of 25 patients with central precocious puberty

Patient No.	Sex	Initial Clinical Presentation	age	Tanner Stage	Bone age Advance (years)	LH		FSH		EST Level	Testosterone Level
						Basal	After test	Basal	After test		
Family A											
A-I	F	Thelarche	5y	3	7	0.8	-	3.8	-	32	-
A-II	F	Thelarche	22m	2	4	2.3	9.1	4.2	15	24	-
Family B											
B-I	F	Thelarche+Menarche	8 y	4	10.5	2.1	-	2.6	-	36.1	-
B-II	F	Thelarche+Menarche	8 y	4	10.5	2.4	-	3	-	40	-
B-III	F	Thelarche	8.5 y	2	11	1.5	-	4.6	-	24.2	-
Family C											
C-I	F	Thelarche	4y	3	5.2	1.2	8.1	3.1	8.4	42	-
C-II	M	Testicular enlargement	7y	2	9	2.1	-	6	-	-	116
Family F											
F-I	F	Thelarche	6.2y	2	8.5	1.8	-	4.2	-	36	-
F-II	F	Thelarche	5.3y	2	8	2.6	-	4.1	-	42	-
Family G											
G-I	F	Thelarche	6.5y	2	9	2.3	-	6.1	-	26	-
G-II	F	Thelarche	4.2y	2	6.4	4.1	-	5.9	-	32	-
Family H											
H-I	F	Thelarche+Menarche	6y	4	10.5	3.2	-	6.1	-	42	-
H-II	F	Thelarche+Menarche	7y	4	11	2.9	-	5.4	-	36	-
Family I											
I-I	F	Thelarche	4y	2	6.2	1.8	-	3.2	-	26	-
I-II	F	Thelarche	5y	2	7.1	2.1	-	2.61	-	30	-
Family K											
K-I	F	Thelarche	7y	2	9.5	2.1	-	6.2	-	22	-
K-II	F	Thelarche	7y	2	10	3.8	-	8.1	-	41	-
Family L											
L-I	F	Thelarche	5Y	2	8.1	1.2	6.9	3.1	17.9	46	-
L-II	F	Thelarche	6.5Y	3	9.5	2.8	8.1	4.8	15.3	31	-
Family M											
M-I	F	Thelarche	6	2	8	2.3	-	4.1	-	26	-
M-II	F	Thelarche	5	3	7.5	1.4	-	3.6	-	28	-
Family N											
N-I	F	Thelarche	7	3	9.5	3.6	-	4.1	-	41	-
N-II	F	Thelarche	7	3	8.7	2.8	-	3.9	-	31	-
Family O											
O-I	F	Thelarche	7	2	9.2	1.4	-	3.1	-	28	-
O-II	F	Thelarche	5.5	3	7	1.2	-	4.9	-	32	-

## Results

**Fig. 1:**
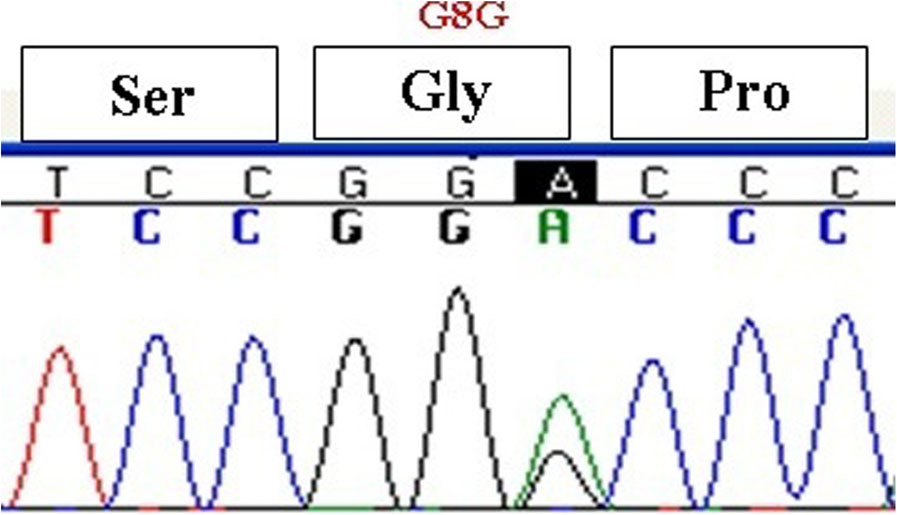
The synonymous polymorphism changing A to G at nucleotide 24 (rs10407968)

**Fig. 2:**
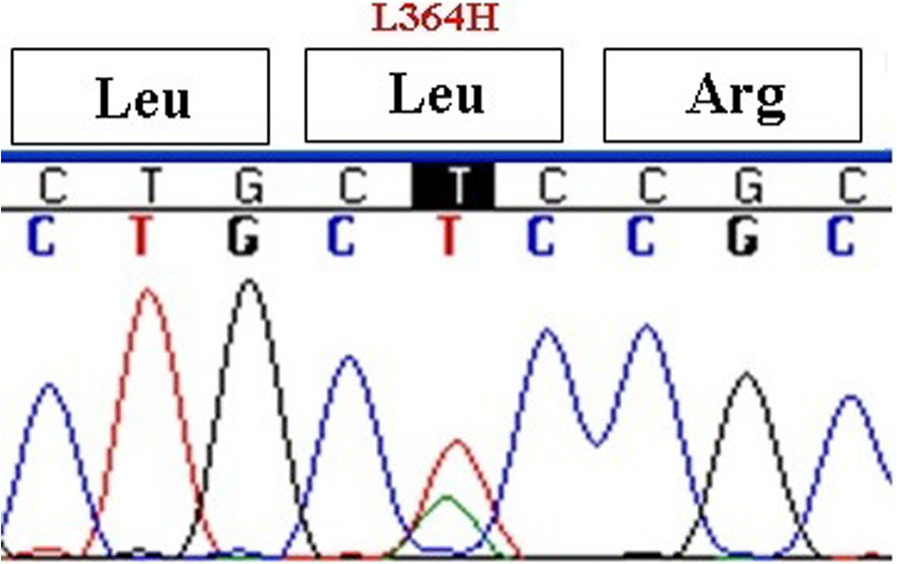
Heterozygous substitution of A for T at nucleotide 1091 in exon five

**Fig. 3:**
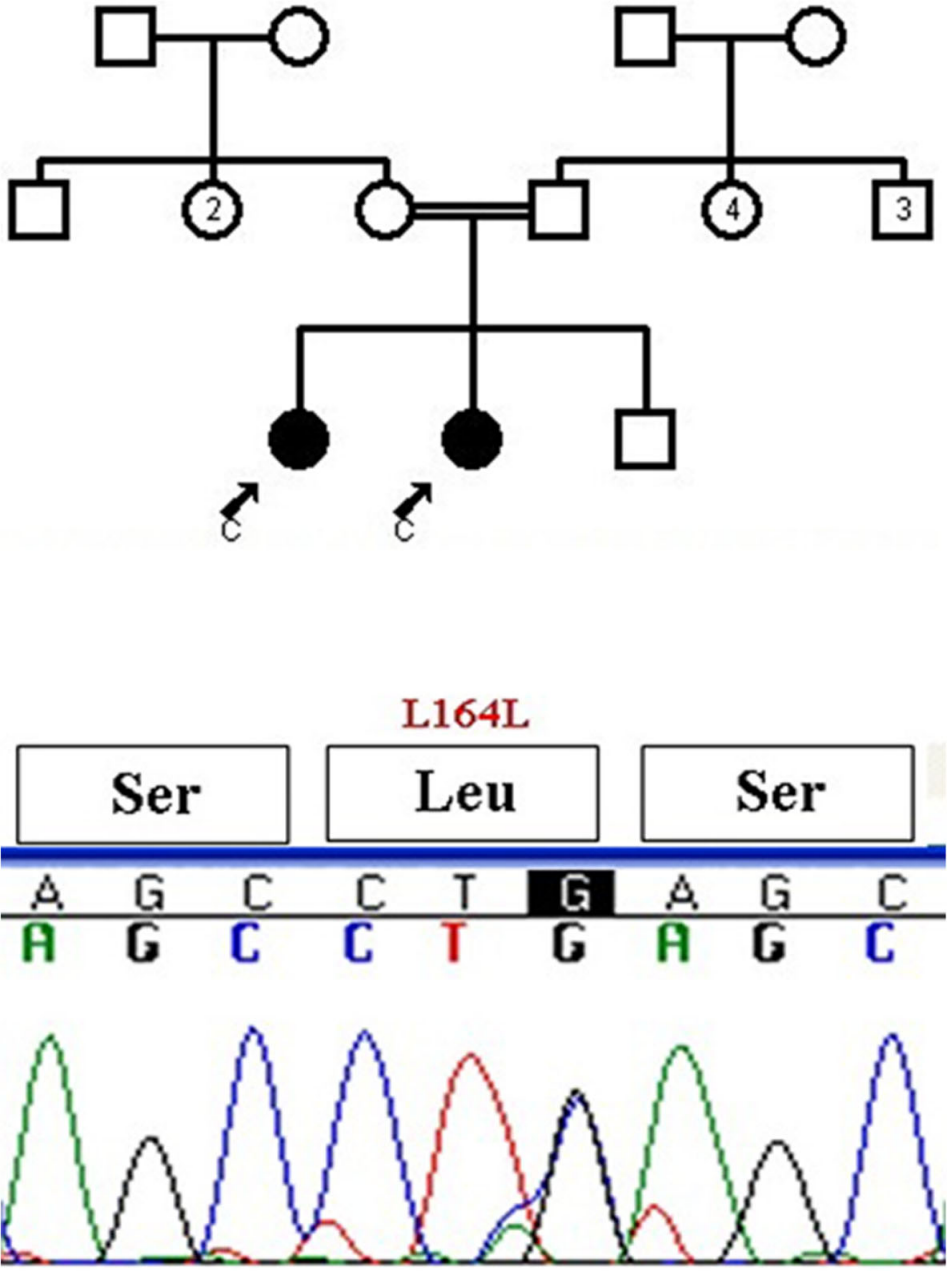
Synonymous 492 C to G SNP was detected for the first time as a novel polymorphism

The mean age of our subjects at diagnosis was 5.76 ± 1.2 years old. Two subjects were twins, and two females first menstruated at 5 and 7 years old. Most subjects had siblings with precocious puberty and the rest of them had family histories of CPP in the first cousins. Height and weight at diagnosis was above the 75th percentile for age, and bone age was advanced in all subjects (7.2 ± 1.5 year). All of the girls had breast and hair development with mean Sexual Maturity Ratings (SMRs) at stages of 2–3. In 23 subjects, mean LH and FSH concentrations were 6 ± 1.3 mIU/mL and 9.5 ± 2.2 mIU/mL, respectively. The GnRH stimulation test was performed in two subjects, and FSH and LH were 29 and 7.8 mIU/mL, respectively. Sonographic findings were compatible with puberty in all subjects. Although, there wasn’t any mutation in *GPR54*, three different SNPs were detected. The synonymous polymorphism changing A to G at nucleotide 24 (rs10407968) was observed in 52% (13/25) of the subjects (Fig. 1). Rs3050132 is also a heterozygous substitution of A for T at nucleotide 1091 in exon 5, resulting in substitution of leucine to histidine at codon 364 which was observed in 64% (16/25) of the cases (Fig. 2). Synonymous 492 C to G SNP was also detected for the first time as a novel polymorphism in one subject (Fig. 3). Generally, SNPs were identified in 22 subjects (88%) and 8 out of these 22 cases (32%) had two different SNPs. No SNPs were detected in exons 3 or 4.

## Discussion

Gonadotropin-dependent CPP is caused by early maturation of the hypothalamic pituitary gonadal axis, which mimics physiological pubertal development at younger than normal ages and leads to the development of secondary sexual characteristics, acceleration of linear growth, advanced bone age, premature epiphyseal closure, and short stature [14]. The condition occurs 5–10-fold more frequently in girls than boys and most sexual precocity in girls is idiopathic [15]. The fact that family members share similar patterns of puberty and ages of onset suggests that genetic factors have roles in the pathogenesis of the pubertal process [16]. Pubertal timing is strongly influenced by genetic, however no single involved gene has been detected. The genetic regulation of pubertal onset most likely results from the additive effect(s) of multiple genes, but monogenic causes of idiopathic CPP likely exist, as cases of familial ICPP have been reported. Mutations in KiSS1 and KiSS1R affect puberty onset and GnRH secretion, and could cause monogenic ICPP [17]. Although, GnRH is the primary hormone responsible for the onset and progression of puberty, genetic factors are also important. To date, genes associated with abnormal pubertal development have been identified, and most of these result in isolated hypogonadotropic hypogonadism [6, 7, 18–24], but they are also considered to be candidate genes in which certain mutations cause precocious puberty. To date few mutations in KiSS1 and *GPR54* have been confirmed as causes of central precocious puberty [4, 22–25]. In present study *GPR54* was examined for genetic variations in 25 Iranians with familial CPP. Although, there were not any mutations in *GPR54*, three different polymorphisms were detected. The rs10407968 (24 A > T) was found in 52% of subjects, rs3050132 (109 T > A) was found in 64% of the subjects, and a novel SNP (492 C > G) was found in one subject. Rs10407968 (c.24A > G) and rs3050132(c.1091 T > A) polymorphisms have been identified in other ethnic groups [20], whereas the c.492C > G (p.L164 L) has not been previously reported. The KO JM et al. study of 101 Korean girls with central precocious puberty revealed four polymorphisms. Two of these, C.196 C > T in GNRH1 and C.546 T > C in GNRH2, were novel [2]. Another report from Korea on non-familial girls showed seven polymorphisms and a (c.1091 T > A) missense mutation. This missense variant induces amino acid substitution of p.Leu364His [26]. We found polymorphisms in 88% of our subjects. We think the rate of polymorphism in familial precocious puberty may be increased. To our knowledge, this study was the first evaluation of familial CPP and also the first study of CPP in the Iranian population. Our subjects were limited in number because we selected patients with CPP who had a sibling or first cousin with precocious puberty. In the Luan study of 272 Chinese girls with central precocious puberty, eight polymorphisms were identified and only one of these (12.5%) was novel [27], but novel polymorphism found in our study was 30% of all detected polymorphisms which may be due to the familial histories.

## Conclusion

The *GPR54* gene has a key role in the puberty process. It has been hypothesized that most of mutations in this gene are lethal; consequently we expected to find no mutations in *GPR54*. We identified SNPs in 88% of the subjects in this study, and 32% had two polymorphisms. It is likely a relationship exists between the SNPs and *GPR54* gene function, affecting familial central precocious puberty. This hypothesis needs further investigation on a larger sample size.

## Abbreviations

CPP: Central precocious puberty
FSH: Follicle stimulating hormone
GnRH: Gonadotropin-releasing hormone
ICPP: Idiopathic central precocious puberty
IRA: Immune Radiometric Assay method
LH`: Luteinizing hormone
ORF: Open reading frame
PCR: Polymerase chain reaction
SNPs: Single Nucleotide Polymorphisms
